# Traumatic avulsion of kidney and spleen into the chest through a ruptured diaphragm in a young worker: a case report

**DOI:** 10.1186/1752-1947-1-178

**Published:** 2007-12-12

**Authors:** Stamatiou Konstantinos, Ilias Georgios, Chlopsios Christos, Karanasiou Vasilissa, Kavouras Nikolaos, Lebrun Fred, Heretis John, Sofras Frank

**Affiliations:** 1 Surgery department, General hospital of Thebes, Thebes, Greece; 2 Urology department, General hospital of Thebes, Thebes, Greece; 3 Urology department, University hospital of Heraclion, Heraclion, Greece

## Abstract

**Introduction:**

Rupture of the diaphragm is almost always due to major trauma. Diaphragmatic injuries are rare (5–7%), usually secondary to blunt, or more rarely to penetrating, thoracic or abdominal trauma. No single investigation provides a reliable diagnosis of diaphragmatic rupture when a patient first arrives at hospital. Almost 33% are suspected on initial chest x-ray, but the percentage is lower in patients who are immediately intubated. Mortality in patients with diaphragmatic rupture following blunt abdominal trauma is generally associated with coexistent vascular and visceral injuries that could be rapidly fatal. It's mandatory that the right diagnosis is reached as soon as possible given that mortality is influenced by the time elapsing between trauma and diagnosis.

**Case presentation:**

A 35-year-old worker was hit by a heavy object while working in the factory. He was transferred immediately to our emergency room. Chest x-ray showed massive left hemothorax without any additional signs to suggest diaphragmatic injury. It was decided to perform immediate surgical exploration before further radiological examination. During surgery, the right kidney and liver appeared normal, but the left kidney and spleen were not found in their anatomical position. The left hemidiaphragm had a10-cm oblique posterior tear. The left kidney was found lacerated in the left side of the chest, separated completely from its vascular pedicle and ureter, along with the entire spleen which was also separated from its vascular tree.

**Conclusion:**

The avulsion of both kidney and spleen following abdominal trauma is uncommon and survival depends on prompt diagnosis and treatment.

## Introduction

In most reported cases diaphragmatic injuries are secondary to blunt, or more rarely penetrating, thoracic or abdominal major trauma, while isolated injuries of the diaphragm rarely occur in patients with blunt trauma [1,2]. Since no single investigation provides a reliable diagnosis of diaphragmatic rupture on arrival at hospital, the diagnosis is frequently missed or delayed unless co-existing associated injuries demand immediate intervention [3]. The occurrence of associated injuries in patients with blunt trauma is variable and depends on the nature of the causative mechanism [4]. Survival depends on prompt diagnosis and treatment. While herniation of adjacent organs through a diaphragmatic tear is not rare, the avulsion of both kidney and spleen following abdominal trauma is very uncommon. The authors describe a case of traumatic avulsion of kidney and spleen into the chest through a ruptured diaphragm and present unique pathological figures. In addition the authors critically evaluate the present evidence regarding ruptured diaphragm and its treatment.

## Case presentation

**Figure 1:**
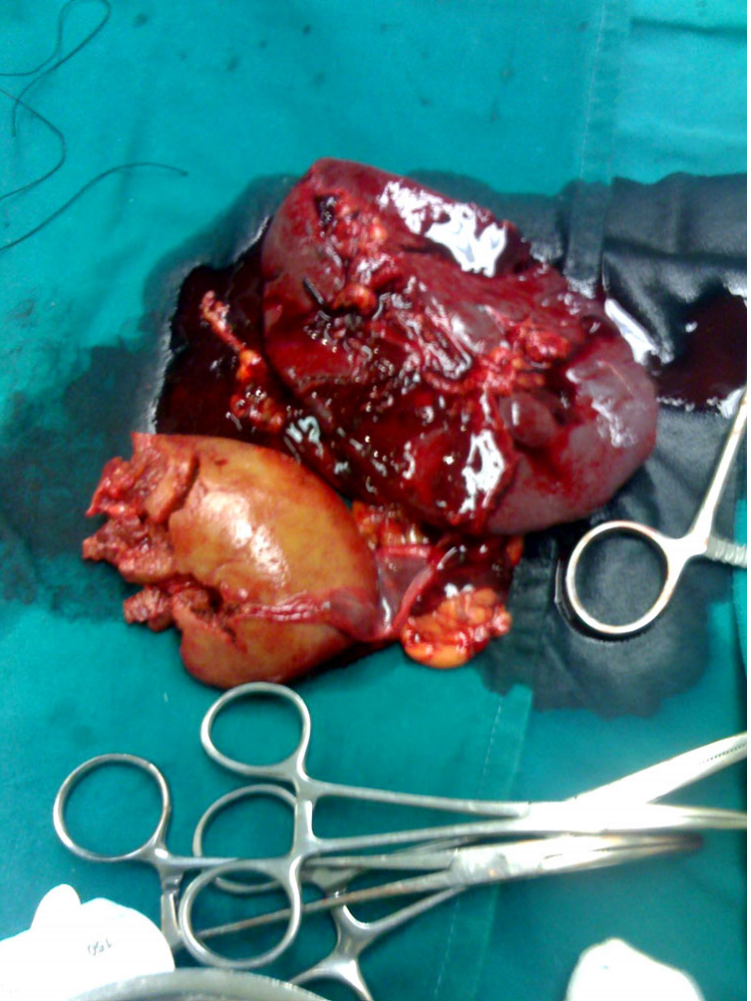
Left kidney, found lacerated in the left chest separated completely from its vascular pedicle and ureter, and spleen, which was also separated from its vascular tree.

**Figure 2:**
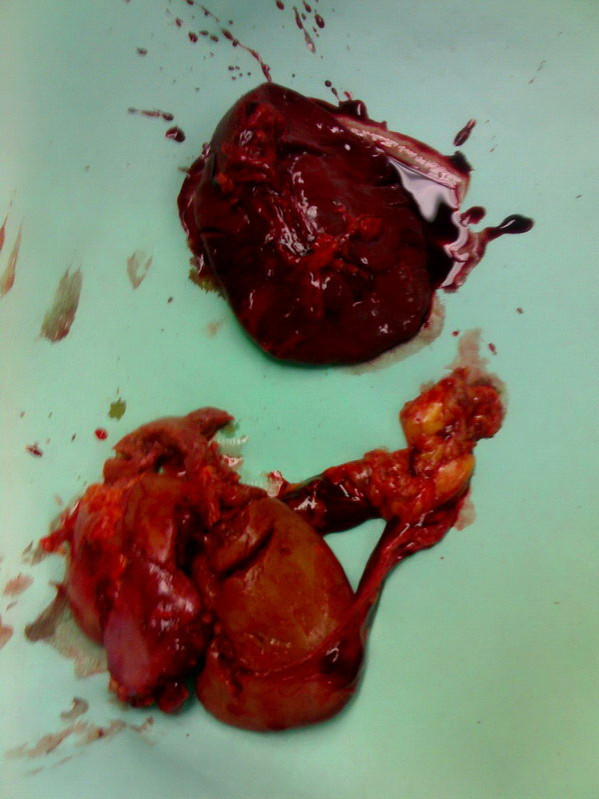
Another view of the left kidney and spleen.

A 35-year-old worker was hit by a heavy object while working in a factory. He was transferred immediately to our emergency room. On arrival his Glasgow Coma Scale was 15/15, blood pressure was stabile (120/80 mmHg) and pulse rate was 86 beats/min. Physical examination revealed multiple rib fractures in the left half of the thorax, abdominal breathing and a right flail chest, macroscopic haematuria and swelling of the left abdominal wall. Chest examination disclosed reduction in breath sounds on the left side. Left upper quadrant abdominal tenderness was evident. A left sided hemothorax was treated by tube thoracostomy, which drained 500 ml of blood initially and 200 ml in the next 30 minutes before surgical exploration was performed. Serial hematocrit ranged between 39–33% over a one hour interval. Chest x-ray showed massive left hemothorax without any additional signs to suggest diaphragmatic injury. When haemorrhagic shock developed (a rapid pulse rate increase to 120 to 130 beats/min and blood pressure decrease to 90/50 mmHg), immediate surgical exploration was performed before further radiological examinations. During surgery, through a midline umbilical abdominal exposure, the right kidney and liver appeared normal, but the left kidney and spleen were not found in their anatomical position. The left hemidiaphragm had a10-cm oblique posterior tear. The left kidney was found lacerated in the left chest, separated completely from its vascular pedicle and ureter, along with the entire spleen which was also separated from its vascular tree. (figures 1 &2)

The renal vessels, the left ureter and renal pelvis were easily identified and ligated separately. The stump of the renal artery did not bleed actively at the time of surgery. The splenic artery and the short gastric arteries were ligated also. The lacerated diaphragm was sutured by single layer non-absorbable sutures. An abdominal tube was placed in the left retroperitoneum at the anatomic place of the left kidney in order to monitor possible bleeding. The patient was hospitalised in the intensive care unit for the next 10 days, recovered uneventfully and was discharged on the 21th postoperative day.

## Discussion and Conclusion

Traumatic rupture of the diaphragm is no longer uncommon. Because of the increasing frequency of motor vehicle accidents, the rate of blunt trauma to the chest and abdomen, which are the most common causes of diaphragmatic rupture, has increased as well [3]. The most frequent mechanism of diaphragmatic injury following blunt trauma is a lateral impact, which distorts the chest wall and shears the diaphragm [3]. Ruptures tend to occur at the central tendon or at the boundary between the tendinous and muscular parts of the diaphragm. In blunt trauma rupture occurs on the left in 65–85% of patients, on the right in 15–35% and bilaterally in 1–12% [4,5]. This is due to the protection offered by the liver on the right, under-diagnosis of rupture on the right, and weakness of the left hemidiaphragm at points of embryonic fusion of the pleuroperitoneal canal.

Isolated diaphragmatic disruptions in patients with blunt trauma rarely occur, accounting for 0.8%–8% of all cases [1]. In most cases traumatic ruptures of the diaphragm occur with associated vascular and visceral injuries [6]. The occurrence, the anatomic location and the severity of such injuries is variable and depends on the nature of the causative mechanism. Common associated injuries include pneumohemothoraces and rib fractures (90%), pelvic fractures (40%–55%), splenic (60%), liver (38%), and renal injuries (10%) [7]. Herniation of adjacent intrathoracic and intra-abdominal organs through a diaphragmatic tear is a relative rare phenomenon and depends on the length of the diaphragmatic injury, the close relationship and the anatomic position. Abdominal injuries most commonly associated with herniation of adjacent organs into the chest cavity are those of spleen and kidney [7]. On the contrary, injuries to the right hemidiaphragm following blunt trauma, are rarely associated with herniation of adjacent organs possibly due to a buffering effect of the liver on the right hemidiaphragm [8]. In 52%–80% of patients with herniation of intra-abdominal organs through diaphragmatic tears, the severity of associated injury is low; low grade renal contusion associated with bowel herniation through a ruptured left hemidiaphragm into the chest is much more common than complete avulsion of solid organs [5]. Only four cases of an entire kidney avulsion into a thoracic cavity and three cases of spleen avulsion have been reported up to date [6,8-12] while avulsion of both spleen and kidney in the thoracic cavity is extremely rare [8].

Radiologic signs which suggest diaphragmatic disruption include abnormally elevated diaphragm, unclear diaphragmatic borders and abnormal gas pattern on plain x-rays [13]. In our patient, however, the simple chest x-ray showed only massive right hemothorax without any additional signs to suggest diaphragmatic injury. Unfortunately, the accuracy of radiographs in the diagnosis of diaphragmatic injuries is insufficient accounting for only 24% for right sided injuries and for 60% for the left-sided injuries [14]. Only 33% of diaphragm ruptures are suspected on initial chest x -ray, while the percentage is lower in patients who are immediately intubated [7]. Because of the lack of sensitivity and specificity of simple imaging modalities, the diagnosis of diaphragmatic disruption usually occurs during the radiologic investigation of the associated injuries [1].

In our case the diagnosis of diaphragmatic disruption occurred during the investigation of the suspected renal injury as our patient presented with gross haematuria, possibly because of the renal laceration. Other cases have reported avulsion of an intact kidney into the thorax however these were not accompanied by macroscopic or microscopic haematuria [7,12]. In the other reported cases of traumatic avulsion of an intact kidney into the thorax there was absence of signs of retroperitoneal hematoma, while hemothorax was massive (1200–1700 ml) [7,12]. In our case, the retroperitoneal hematoma was visible, while hemothorax was limited, indicating severe retroperitoneal bleeding and less migration of blood into the chest through the ruptured diaphragm.

The accuracy of CT in the diagnosis of diaphragmatic injuries is higher, being 88% for left-sided injuries and 70% for right-sided injuries [15,16]. Still, since many of the diaphragmatic disruptions have a delayed appearance, about 12–14% of cases have a delayed radiologic diagnosis while the remaining cases are diagnosed at laparotomy or thoracotomy [6]. Shanmuganathan et al [17] found MR imaging reliable in the diagnosis of traumatic diaphragmatic injury when CT and chest radiographic findings were questionable. Unfortunately, many trauma patients in the acute setting require support devices that are not compatible with MR imaging.

The development of endoscopic techniques allows the use of thoracoscopy and laparoscopy for the evaluation and treatment of hemodynamically stable patients with abdominal trauma. The general contraindications refer above all to the state of haemodynamic instability of the patient and to seriously ill patients (ASA IV) [18]. In the absence of any specific contraindications for the specific laparoscopic procedure to be carried out, many abdominal traumas requiring immediate surgery exploration can now be diagnosed with the laparoscopic approach [19]. Indeed, both thoracoscopy and laparoscopy have been demonstrated as feasible and safe methods to confirm diaphragmatic disruptions in selected patients [3,14,20].

The difficulties in diagnosing traumatic diaphragmatic rupture on admission are the most common causes of morbidity and mortality. Traumatic diaphragmatic hernia is incorrectly diagnosed in up to 33% of cases during the immediate post-traumatic period [21], while during the acute phase the diaphragmatic rupture may be missed because of shock, respiratory insufficiency or coma [16]. Mortality in patients with diaphragmatic rupture following blunt abdominal trauma is generally associated with coexistent vascular and visceral injuries that could be rapidly fatal, but it is also associated with acute respiratory compromise from visceral strangulation. Indeed, diaphragmatic hernias associated with severe injuries have been linked with mortality rates of 52%–100% [5] while mortality as high as 25–60% has been reported in patients with coexistent untreated strangulation of incarcerated viscera [22].

It's mandatory that the right diagnosis is reached as soon as possible given that mortality is mostly influenced by the time elapsing between trauma and diagnosis. Therefore selection of the most appropriate radiological technique is important for the accurate diagnosis of traumatic diaphragmatic at the time of admission. To render the appropriate diagnosis, the radiologist must be familiar with the varied imaging manifestations of injury, and maintain a high index of suspicion within the appropriate clinical setting. In the case of clinical suspicion, when radiological signs are lacking, there should be a low threshold for using further imaging to assess the diaphragm; either open surgery or endoscopic procedures should be performed immediately in order to diagnose and rule out the injury. Treatment choice is mainly based on the clinical situation therefore the timing of these procedures should be in accordance with the hemodynamic and respiratory status of the patient. Endoscopic procedures can be used when there are no other indications for immediate surgical intervention thereby reducing the number of nontherapeutic laparotomies and thoracotomies performed.

## Competing interests

The author(s) declare that they have no competing interests.

## Authors' contributions

SK, IG and CC were involved in the case directly. SK drafted the manuscript. LF and KV took part in the care of the patient and contributed in the preparation of the manuscript. HI contributed in carrying out the medical literature search. SF was involved in conception of the article and revising it critically for important intellectual data before final approval. All authors reviewed the final drafting of this manuscript.

## Consent

The authors would like to thank the patient for providing informed consent for the publication of this case report.
